# Liposuction and port site nipple sparing mastectomy: an alternative method for the operative treatment of gynecomastia at Alexandria main university hospital

**DOI:** 10.1186/s12893-023-02146-9

**Published:** 2023-08-21

**Authors:** Mohamed Asal, Moataz Ewedah, Mahmoud Bassiony, Ahmed Abdelatif

**Affiliations:** 1 Alexandria University Faculty of Medicine, Alexandria, 21521 Egypt; 2 Barking, Havering and Redbridge University Hospitals NHS Trust, Essex, UK

**Keywords:** Gynecomastia, Surgical intervention, Minimally invasive techniques, Liposuction, Mastectomy, Recurrence rate, Complication rates, Patient satisfaction

## Abstract

**Background:**

Gynecomastia is characterized by unusually large masses that radiate concentrically from the base of the nipple and is caused by abnormal growth of the glandular tissue of the male breast. An alternative strategy for the surgical treatment of gynecomastia was used in this experimental study, which aims to use liposuction and port site nipple sparing mastectomy.

**Methods:**

The study was conducted in the surgical oncology unit at Alexandria Main University Hospital included 103 patients with a mean age of 27 and no medical history. 100 patients had bilateral gynecomastia, and three patients had unilateral gynecomastia,with two having it on the right side and one on the left.

**Results:**

Among the 103 participants, 83 had grade II gynecomastia and 20 had grade I. Only one of the three patients who participated in the study had an expanding hematoma on one side that needed to be surgically evacuated in the operating room. None of our patients experienced an infection or seroma following surgery. Furthermore, only three of our patients experienced nipple areolar complicated superficial epidermolysis, which need regular dressings until recovery. Of the 103 patients, 97 (94.17%) were pleased with the outcomes.

**Conclusion:**

Liposuction and port site nipple sparing mastectomy are viable options for treating grade I to II gynecomastia, particularly if the patient prefers a more aesthetically pleasing chest contour; no scars equals better patient satisfaction.

**Trial registration:**

retrospectively registered.

**Supplementary Information:**

The online version contains supplementary material available at 10.1186/s12893-023-02146-9.

## Background

Gynecomastia is a benign proliferation of glandular breast tissue in men [1, 2]. It is common and has a prevalence of 32–65% of the population with peak ages from 13 to 20 [3, 4].

Management of gynecomastia has a variety of options. Asymptomatic gynecomastia can go with just reassurance and follow up, while symptomatic gynecomastia with associated breast pain or tenderness, further evaluation to determine the probable cause of gynecomastia should be undertaken [2, 5].

Medical therapy will probably be ineffective in males with long-standing, symptomatic gynecomastia, and surgery might well be considered. If a patient does not respond to medication therapy, is unable to endure it, or refuses treatment, or wants immediate gynecomastia correction, in addition, if the patient chooses surgery for cosmetic reasons such as social anxiety. Surgery should be considered for this type of patients [2, 5]. Suction lipectomy or glandular breast tissue removal through a periareolar incision are two current standard surgical techniques [2, 6]. Several clinical studies were conducted to compare the superiority of each technique in terms of esthetic results and complications [6–9].

The postoperative shape and symmetry of the nipple areolar complex (NAC) were not as satisfactory as postoperative breast size and symmetry [8]. Furthermore, they demonstrated that in patients with Simon grades I to IIa gynecomastia, subcutaneous mastectomy with a circumareolar incision and no additional liposuction provides a good or very good aesthetic outcome, however, achieving a very good or even a good aesthetic outcome in patients with Simon grades IIb to III gynecomastia is difficult [8]. While liposuction patients have better cosmetic outcomes and fewer complications in comparison with patients who chose mastectomy [7]. Although these two combined procedures are the most common, the breast tissue can be removed by a subcutaneous approach, but there is a risk of consequences such as saucer-like deformity, nipple necrosis, and contour irregularity that must be considered [10, 11].

Lipectomy with subcutaneous mastectomy inevitably creates a scar on the anterior chest wall, which may have a psychological impact on patients, particularly adolescents. Generally, the cosmetic outcome following conventional surgery is not satisfying to some patients [10, 12, 13]. Thus, there is a tendency towards the use of a much less invasive procedure. In this study we innovated a new modification to the wet liposuction technique of grade 1, 2 gynecomastia patients avoiding any further incisions for excision of the retroareolar disc or remnant tissue after liposuction which results in better cosmetic outcomes.

## Methods

The study employed a prospective single-limb design over one year, from August 1st, 2021 to August 1st, 2022. The study was conducted at the Department of Surgical Oncology, Faculty of Medicine, Alexandria University, and included patients who met the inclusion criteria.

*Inclusion criteria* consisted of patients aged between 19 and 47 years, with no history of hormone or drug use, no history of endocrine-related diseases, and no clinical or radiological evidence of breast lump with gynecomastia grade I or/and II only. The Simon classification system was used to determine the extent of gynecomastia. This system categorizes gynecomastia into four grades, with grade I and II indicating mild-to-moderate gynecomastia that does not require the removal of excess skin [14].

*Exclusion criteria* consisted of individuals with comorbid conditions such as diabetes, chronic renal failure, liver cirrhosis, cardiac and hypertensive patients, grade III or IV patients, patients receiving hormone therapy, athletes who take supplements, and patients with mental instability.

All subjects provided informed consent before participating in the study.

### All study participants underwent the following procedures

Taking a thorough history that includes: Name, age, sex, occupation, address; a specified form was filled out for each patient to record his data; past history of previous interventions; hospital diagnosis; date of admission; medical and past history.

Careful clinical evaluation including blood pressure, temperature, heart rate, and respiration rate, as well as symptoms of (Pallor, Cyanosis, Jaundice, and Lymph node enlargement).

#### Types of interventions

Subcutaneous liposuction combined with lipo-suction-induced glandular excision.

### Technique

General anesthesia is used during the surgical process. In order to cross liposuction tunnels and access the glandular tissue in the periareolar region, markings are made with the patient standing up and include the inframammary fold, boundaries of liposuction, and skin incisions that are 5–7 mm long in the point of crossing of the anterior axillary line and the horizontal line on line with the inframammary fold.

**Fig. 1:**
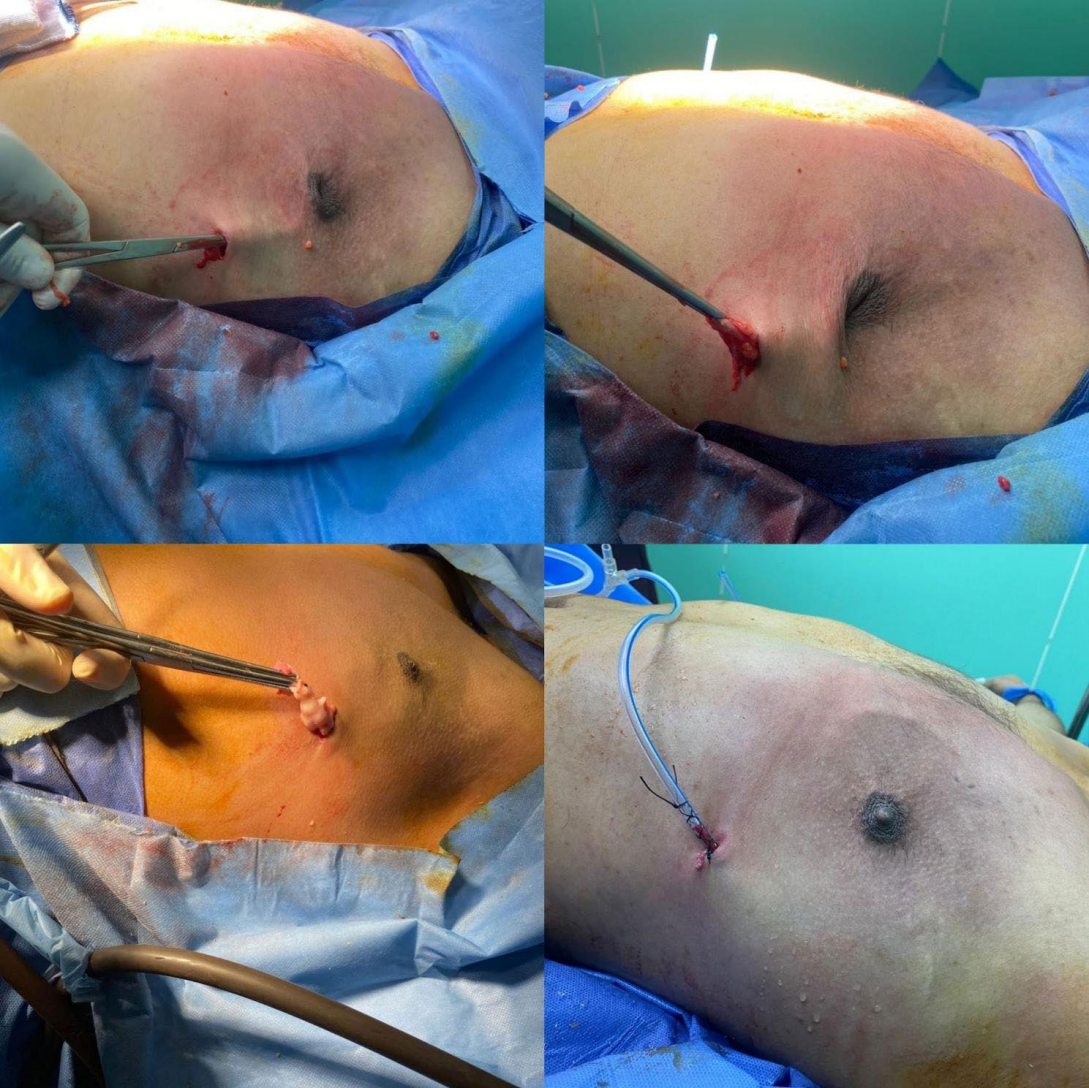
Detailed depiction of the sequential steps involved in our procedure for inframammary liposuction and glandular excision The illustration highlights the meticulous removal of all adipose tissue and the retroareolar disc. Notably, patients presenting with substantial glandular tissue necessitate glandular excision, which is skillfully executed through the same port site

**Fig. 2:**
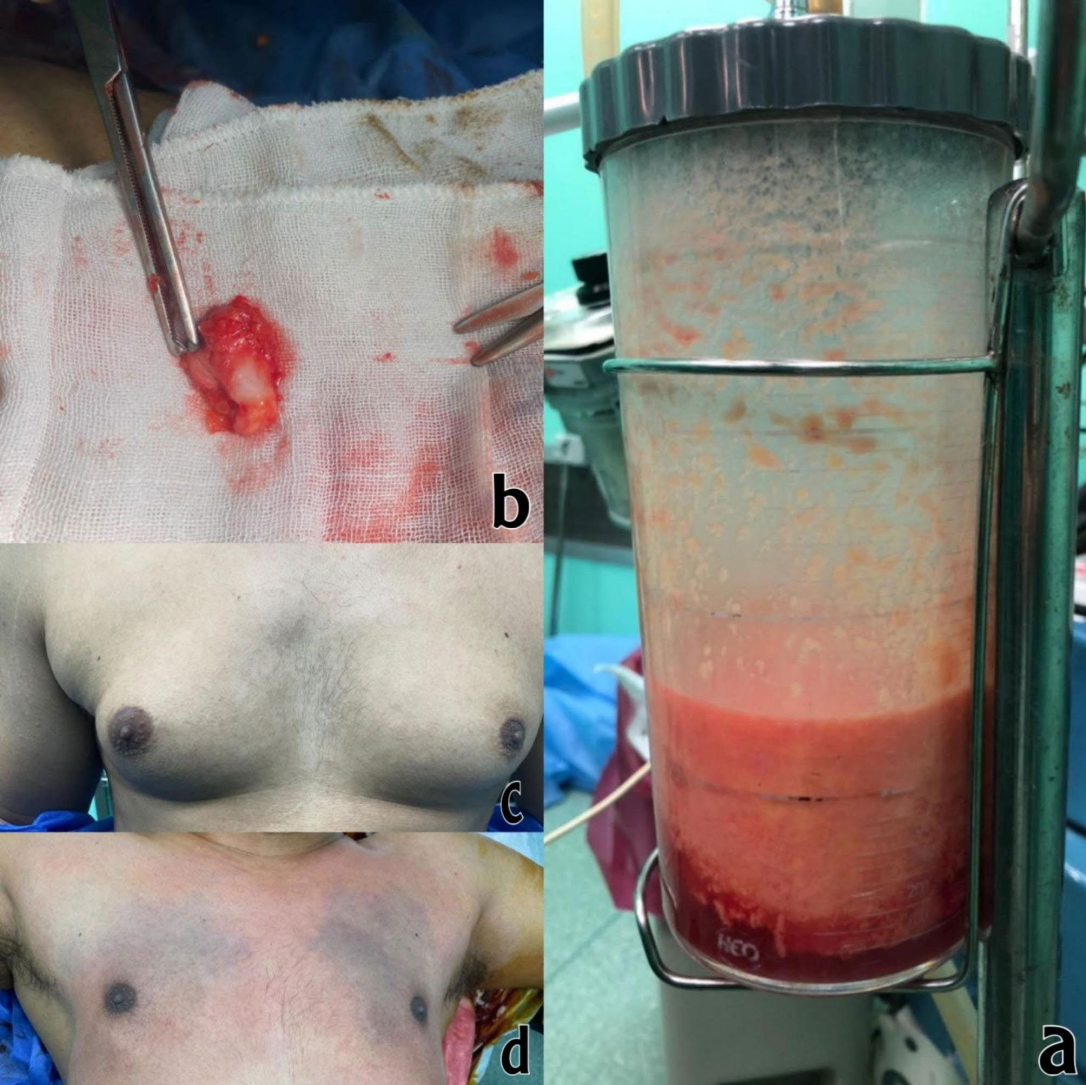
Detailed depiction of the outcomes and components related to the procedure Panel **a** illustrates the extent of liposuction performed, highlighting the areas from which adipose tissue was successfully removed. Panel **b** showcases the size of the gland that was excised during the procedure. Panels **c** and **d** present a comparative view of the pre- and postoperative results, demonstrating the visible changes achieved after the surgical intervention

1. A side arm board was extended carefully to 90 degrees to avoid any traction injury to the arm and then raised up about 15 to 30 degrees.
2. Incision over the inframammary line and anterior axillary line junction.
3. A tumescent technique for liposuction using a solution consisting of a 500 ml IV bottle of sterile physiologic normal saline (0.9% NcCl).
4. One 10 ml of 2% plain lidocaine (200 gm of lidocaine). One 1-ml ampule of 1:1000 epinephrine (1 mg of epinephrine). The volume of injection depended on the size of the breast, usually 500 mL solution per breast was adequate. Rapid sequence injection of the tumescent solution is achieved by maintaining the solution on direct compression by Arm Blood Pressure meter Cuff connected with a 12-gauge, blunt-tipped, multiholed infiltrating cannula to facilitate the maximum rate of tumescent infiltration (Fig. 1).
5. After 15 min waiting, liposuction starts using liposuction cannula either 4,5,6 mm.The suctioned volume is 500 ml +/-150 from each breast.Palpable retroareolar disc or residual fibrous fatty tissue can be palpable for which we start our novel surgical resection technique of nipple sparing mastectomy by clamping the remnant disc or breast tissue through the only made incision and grabbing it to outside the wound leaving point of traction on the NAC or the skin which are cut with long scissor until all adhesions released and retroareolar disc is excised totally. Insertion of redivac drain through the same incision and leaving it for 4 days postoperatively. Chest wall compression bandaging for 7–14 days to maintain compression (Fig. 2).

## Results

Our study included a total of 103 participants with a mean age of 27 years and no significant medical history. The demographic and preoperative clinical data of the patients can be found in Table 1. The average duration of the surgical procedure was 45 min.

**Table 1:** Demographic and preoperative clinical data of the patients

	Studied group (*n* = 103)
Age (years)	
Range	19-47
Mean	27
SD	7
Affected side, n	
Unilateral Right	2
Unilateral Left	1
Bilateral	100
Grade, *n* (%)	
Grade 1	20 (19.4%)
Grade 2	83 (80.6%)

**Fig. 3:**
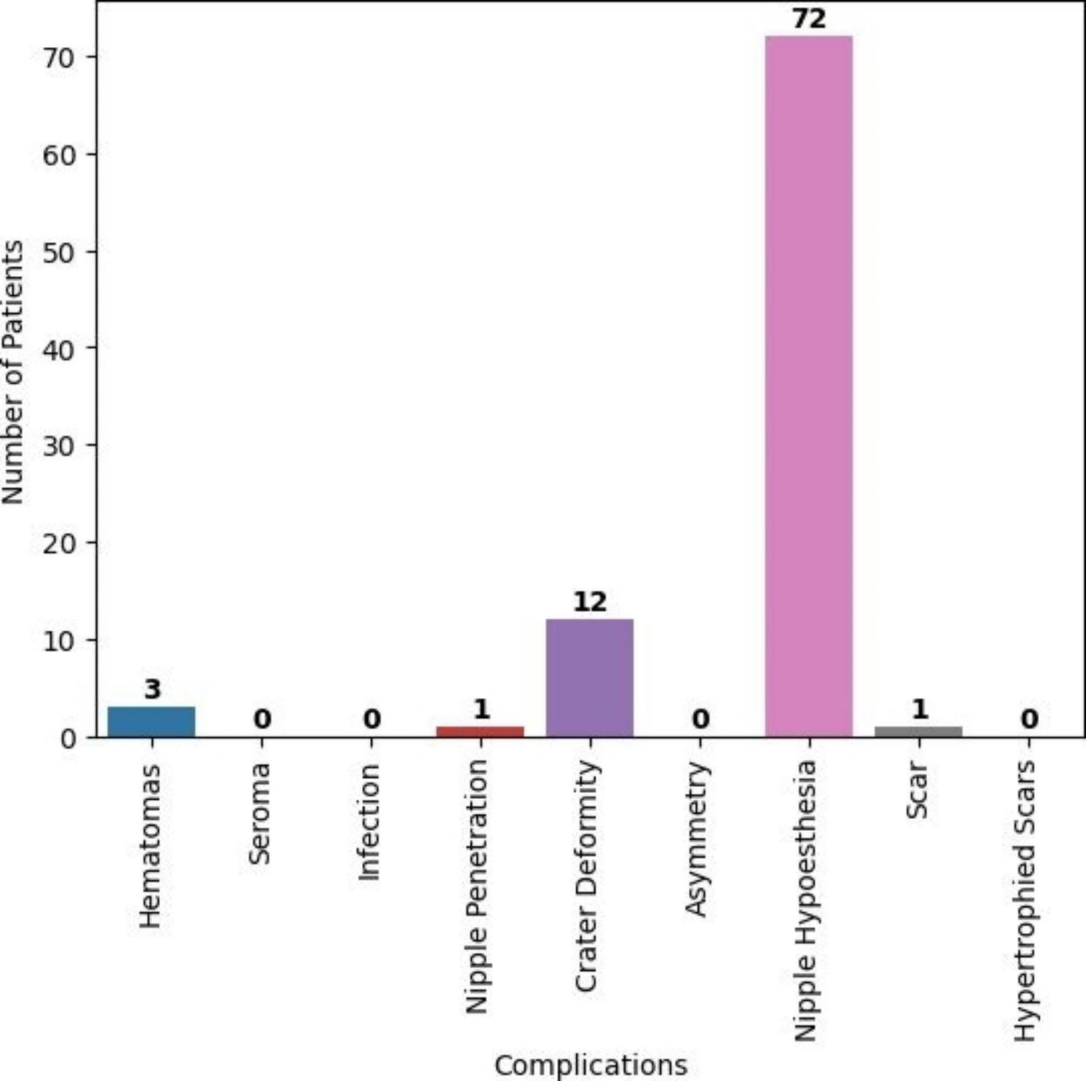
Comprehensive overview of the specific types of complications encountered during the trial period

During the follow-up period, which ranged from 6 to 18 months with an average of 12 months, we observed the following complications: (Fig. 3)

**Fig. 4:**
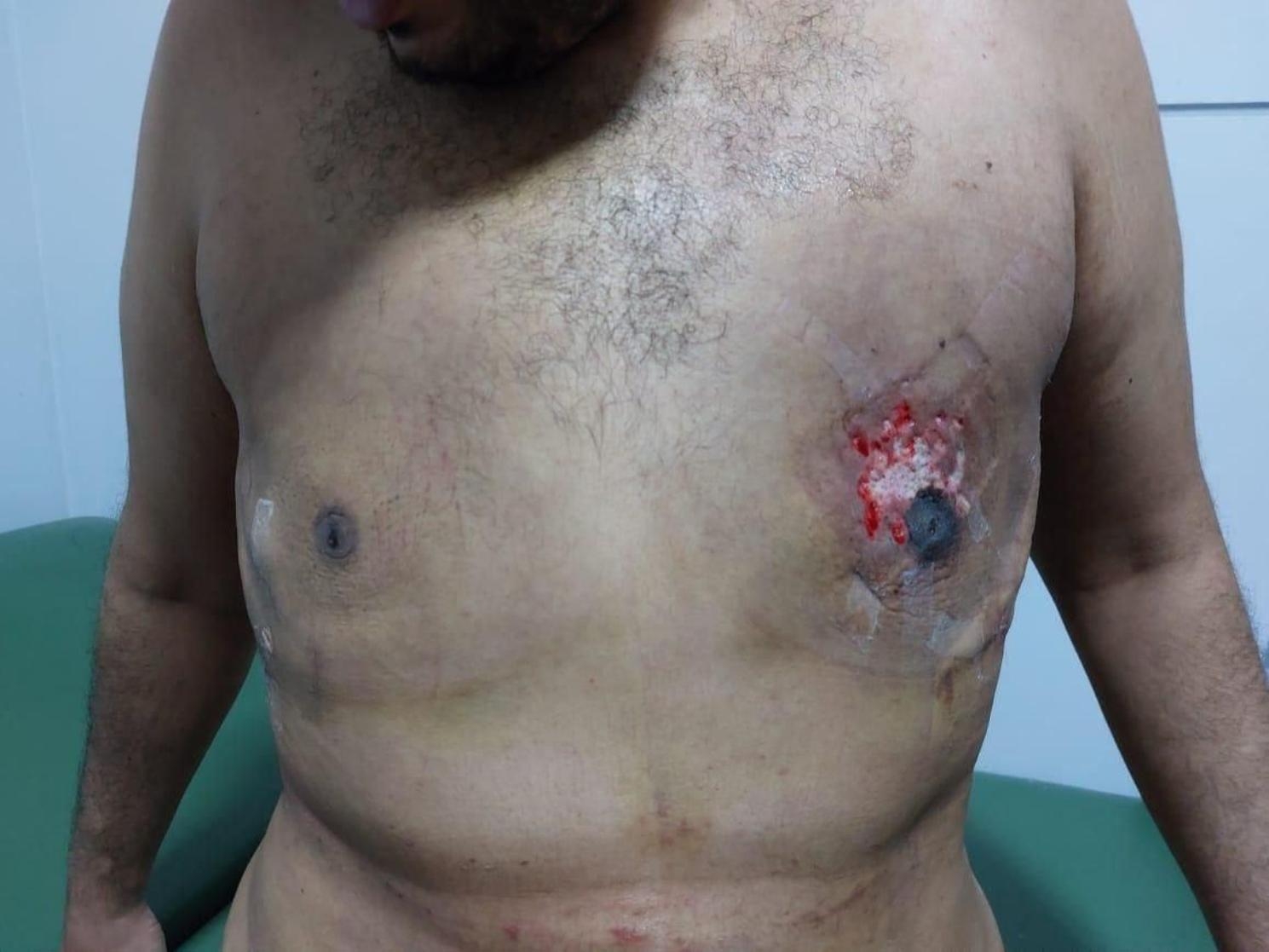
Shows superficial epidermolysis occurring in the nipple areolar complex

1. Hematomas: Three patients experienced hematomas, with one patient having an expansive hematoma on one side that required surgical evacuation through the drain site in the operating room. This occurred within hours of surgery and was observed in a patient with grade II gynecomastia. We noted a correlation between the development of hematomas and the amount of suctioned fluid.
2. Nipple-areolar complex necrosis: Three patients experienced superficial epidermolysis of the nipple-areolar complex, which required frequent dressing until healing. (Fig. 4)
3. Nipple penetration: One patient experienced intraoperative penetration of the areola while removing the retroareolar disc. No further surgical intervention was required.
4. Nipple retraction and soft tissue deformity: To prevent undesirable depressions, it was necessary to leave a thickness of 3–5 mm of retroareolar disc behind the areola in patients with fatty breasts. Twelve patients (11.6%) developed a minor deformity known as crater deformity, but none of them expressed dissatisfaction or sought corrective surgery. None of our patients experienced nipple retraction.
5. Nipple hypoesthesia: During the follow-up period, 72 (69.9%) patients reported nipple hypoesthesia.
6. Scar: There were no visible scars, in contrast to traditional gynecomastia surgery. Only one patient complained about the shape of the scar at the drain site.

Remarkably, none of the patients in our study reported experiencing any asymmetry. Additionally, there were no cases of postoperative infection, postoperative seroma, or hypertrophic scars observed among the patients.

In terms of patient satisfaction, 97 (94.17%) of the 103 patients reported being satisfied with the outcomes of the surgery, as assessed by a satisfaction questionnaire. This questionnaire included questions about any problems with the lateral scar, nipple hypoesthesia, asymmetry, and an overall satisfaction scale. (Additional File 1).

**Fig. 5:**
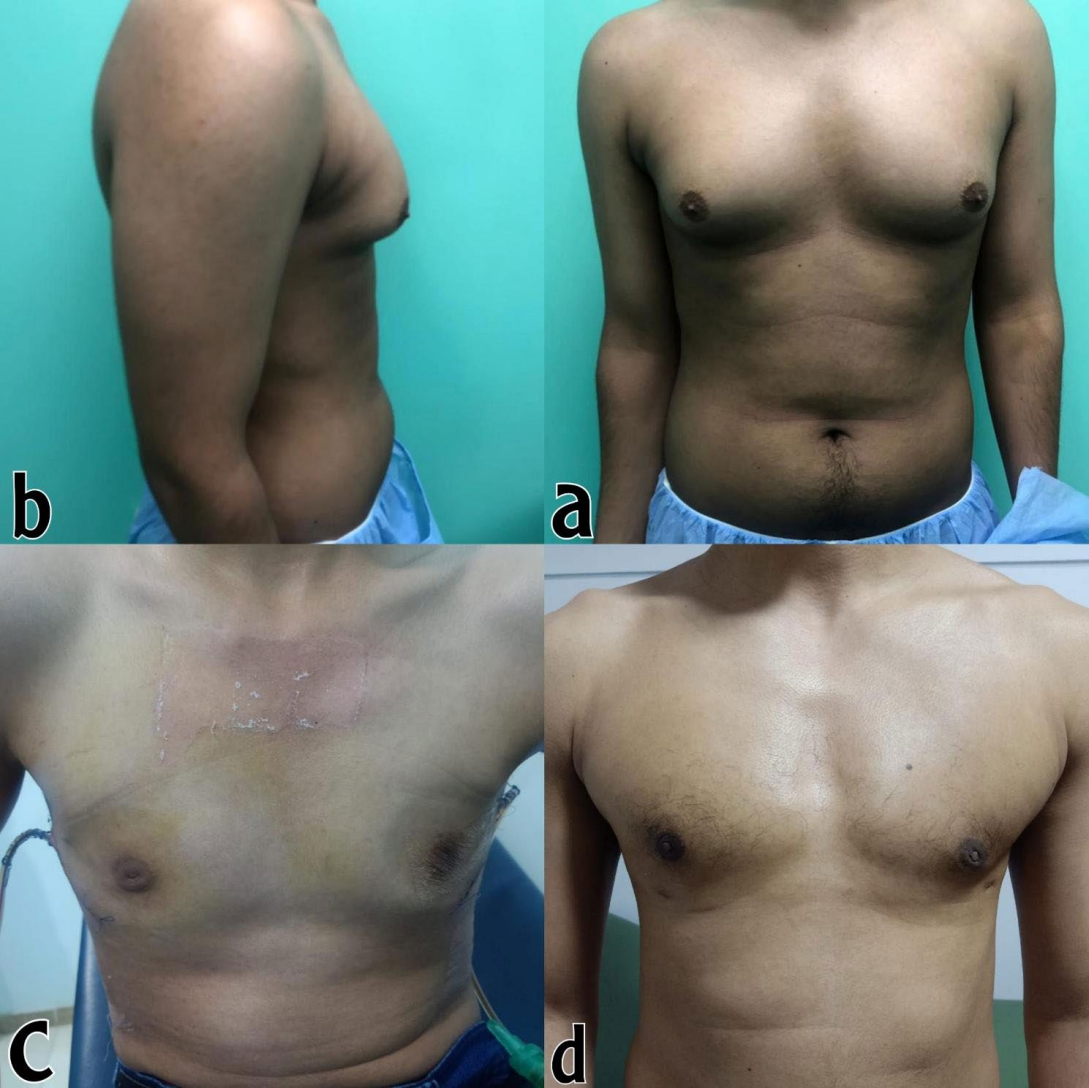
Describes a detailed depiction of the pre-operative and the outcome of the surgery. The figure is divided into four panels, labeled as **a, b, c,** and **d**, each depicting different views of the surgical outcome Panels **a** and **b** provide a visual representation of the condition before the surgery. Panel **c** displays the immediate outcome after the dressing has been applied following the surgery. Finally, panel **d** shows the results after a period of three months post-surgery

**Fig. 6:**
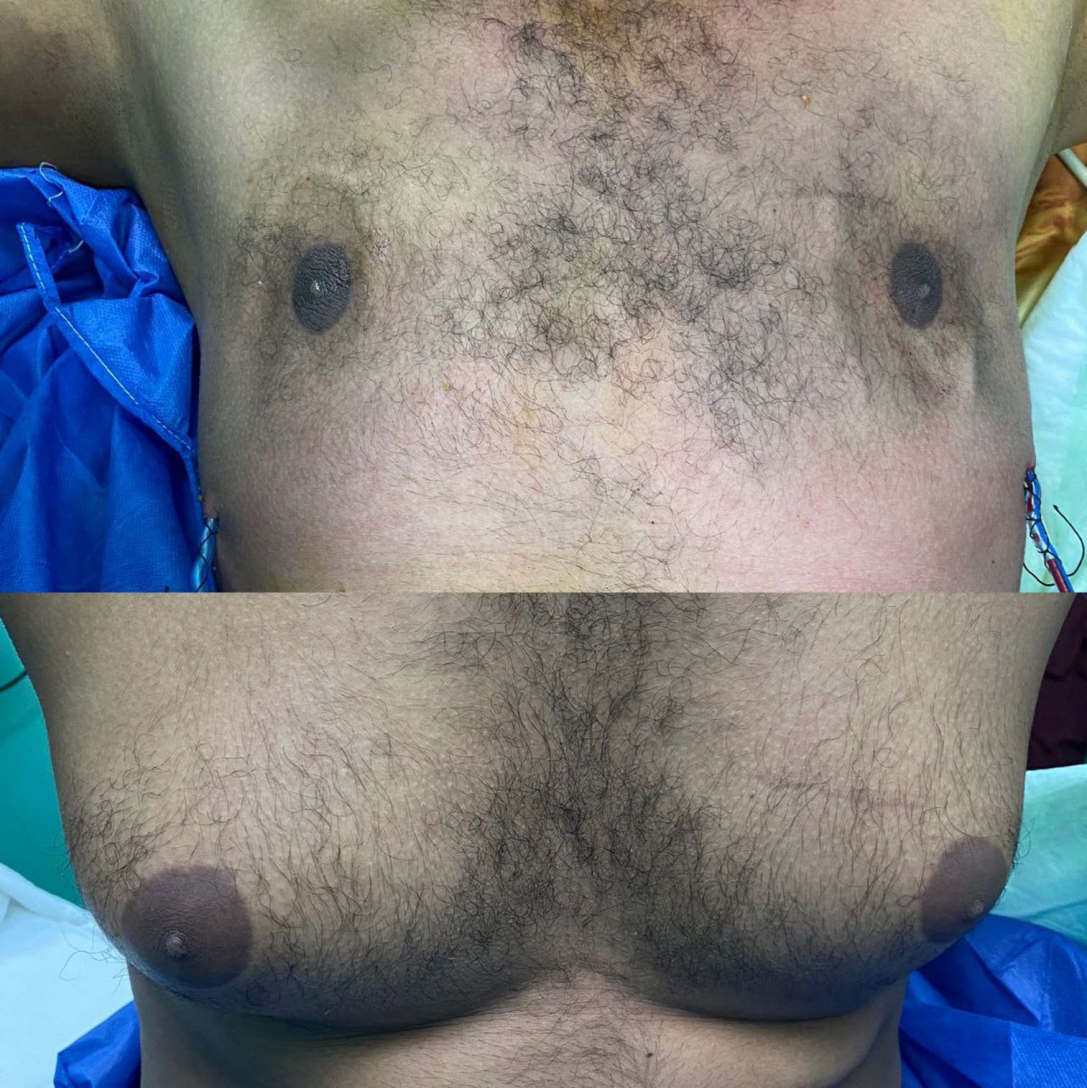
Shows another case before and after surgery

All patients were discharged on the same day of the surgery and were able to resume exercise and normal activities within a few days. The majority of patients returned to work within three weeks after the procedure. Cosmetic differences before and after surgery can be seen in Figs. 5 and 6.

Panels a and b provide a visual representation of the condition before the surgery. Panel c displays the immediate outcome after the dressing has been applied following the surgery. Finally, panel d shows the results after a period of three months post-surgery.

## Discussion

The presence of gynecomastia, characterized by the imbalance between endogenous estrogen and androgen hormones, is a common benign enlargement of the male breast [10, 11]. Psychological stress and the desire for improved cosmetic appearance often lead gynecomastia patients to opt for surgical intervention before the manifestation of pathological changes [14]. Consequently, surgeons have been motivated to explore new, less invasive techniques that can provide satisfactory outcomes for patients [15–21].

Traditionally, there are three surgical methods employed for gynecomastia treatment: conventional lipectomy, subcutaneous mastectomy, and a combination of the two. Various procedures, such as syringe liposuction, suction-assisted liposuction, power-assisted liposuction, and ultrasound-assisted liposuction, have been described as easier to perform compared to other techniques [11, 22]. These methods allow for the creation of a superficial plane between the skin and breast tissue, resulting in no visible scarring on the breast. However, due to their high recurrence rate, they are typically used as a preliminary step before open excision in most cases [23].

In 2005, Hammond and colleagues introduced a novel approach for surgical mastectomy in gynecomastia treatment called ultrasonographically aided liposuction. This technique combines the advantages of both approaches and is suitable for fatty breasts, although scarring remains a concern [11, 24]. In recent decades, other minimally invasive procedures have also made significant advancements. Lipectomy is a necessary component of traditional procedures [25]. However, the technique we have adapted in our study allows for the removal of both breast tissue and fat, thereby reducing the recurrence rate. The disc is removed through the drain site before the insertion of the Redivac drain, resulting in minimal scarring.

The extent and thickness of tissue to be left behind in this procedure vary from patient to patient and need to be assessed by the surgeon. Surgeons face the challenge of striking a balance between removing all the disc tissue, which may lead to nipple retraction, particularly in large fatty breasts, and the possibility of recurrence if the tissue of the retroareolar disc is preserved.

In our study, all patients underwent surgery as a day case procedure, with same-day discharge and oral analgesia for a few days. Compression stockings were applied for 48 h before transitioning to tight clothing. Only a small percentage of our patients developed hematoma, and surgical intervention was required in only one case. The occurrence of seroma or surgical site infection was absent in our patients, which differs from the findings of other authors [26]. The absence of seroma complications in our study may be attributed to the removal of drains after 3–4 days, suggesting that the use of drains may reduce seroma rates. However, it should be noted that the universal standard of care regarding drains after gynecomastia surgery remains unclear, and surgeon practices vary [27, 28].

Nipple areola complex necrosis was reported in three patients who developed superficial epidermolysis. However, none of these patients required corrective surgery. Alessandro Innocenti, et al. have reported 1.92% of nipple areola complex necrosis with aspiration technique [29]. Reoperation rate of liposuction-assisted surgery is between 0.6 and 25% according to Theddeus Octavianus Hari Prasetyono, etc. [30].

In comparison to a study conducted in Egypt [31], our study had a lower incidence of short-term complications such as hematoma, partial necrosis, and seroma. Nipple asymmetry was absent in our patients, whereas previous studies using the liposuction technique reported an 11% occurrence of nipple asymmetry [32]. The suctioned fat volume was consistent between both sides in our patients, with a difference of less than 30 ml.

The challenge encountered when removing the entire retroareolar disc resides in the delicate balance of preserving enough tissue to prevent complications such as nipple necrosis or retraction, while also avoiding retaining excessive tissue that may contribute to recurrence. Based on our expertise, we recommend leaving approximately 5 mm of retroareolar disc tissue to provide adequate support for the nipple areola complex and prevent retraction. It is noteworthy that none of our patients experienced nipple retraction, although a small percentage did report a minor deformity known as crater deformity, which did not necessitate corrective surgery. Additionally, it is worth mentioning that Caridi et al. published a study reporting no cases of nipple retraction with open surgery, as the surrounding subcutaneous tissue was able to provide sufficient support for the nipple areola complex [33]. This finding adds further support to the importance of preserving an appropriate amount of tissue during surgical intervention for gynecomastia.

A systematic review reported varying rates of hypo or hyperesthesia, with aspiration-only techniques resulting in a rate of 23.6% and excision techniques resulting in a rate of 57.4% [16]. In our study, 69.9% of patients reported numbness or hypoesthesia during the follow-up period, but none reported tenderness, chronic pain, or hyperesthesia.

One notable advantage of our technique is the absence of scarring issues. There is no circumareolar scar, and even the scar at the drain site is minimal and located on the anterior axillary line. None of our patients expressed dissatisfaction related to scarring. The cost of our technique is minimal, as we utilized suction technique without additional vibration amplification of sound energy at resonance (VASER) or ultrasound guidance, further adding to its advantages.

The majority of our patients (94.1%) reported satisfaction with the procedure. Even in the group of patients who were not completely satisfied, minor complications were observed, but none of them sought corrective surgery either immediately after the procedure or in the postoperative period.

Limitations of our study should be acknowledged to provide a comprehensive understanding of the findings. Firstly, it is important to note that our study design was a prospective single-limb design over a one-year period. While this design allowed us to gather valuable data on our adapted technique, it may limit the generalisability of our results to a broader population or longer-term outcomes.

Additionally, the sample size of our study was relatively small, which may impact the statistical power and limit the ability to detect rare complications or assess the effectiveness of the technique in specific subgroups of patients. Further studies with larger sample sizes and longer follow-up periods are warranted to validate our findings and explore potential differences in outcomes across different patient populations.

Another potential limitation is the lack of a control group for comparison. Without a control group, it is challenging to ascertain the superiority of our adapted technique over other existing surgical approaches. Future studies incorporating control groups would provide valuable insights into the comparative effectiveness and safety of different surgical methods for gynecomastia treatment.

Furthermore, our study focused primarily on short-term outcomes, with a particular emphasis on complications and patient satisfaction. Long-term follow-up data, including factors such as recurrence rates, long-term cosmetic outcomes, and patient-reported quality of life measures, would provide a more comprehensive evaluation of our adapted technique’s efficacy and durability.

Lastly, it is crucial to acknowledge that our study was conducted at a single center, which may introduce potential biases and limit the generalizability of our findings to other settings or surgical practices. Further multi-center studies involving diverse patient populations and surgical teams would enhance the external validity of our results.

In summary, while our adapted technique for gynecomastia surgery demonstrates promising results in terms of minimal scarring, low complication rates, and high patient satisfaction, it is important to consider the limitations of our study, including the study design, sample size, lack of a control group, short-term follow-up, and single-center nature. Future research efforts should address these limitations to provide a more robust and comprehensive understanding of the effectiveness and long-term outcomes associated with our surgical approach. Overall, our study emphasizes the positive outcomes and patient satisfaction associated with our adapted surgical approach for gynecomastia.

## Conclusion

Grade I to II gynecomastia can be successfully treated with liposuction and port site nipple sparing mastectomy, especially if the patient desires a more aesthetically acceptable chest contour. Patients are more likely to be satisfied with this procedure because there are no scars. Our nipple-sparing mastectomy at the port site with drainage through the same lateral drain site incision demonstrated excellent results and improved upon the prior approaches in terms of aesthetic outcome and patient satisfaction.

### Future research directions and recommendations

- Comparison of different surgical techniques: Further studies are needed to compare the efficacy, safety, and recurrence rates of different surgical techniques for gynecomastia. This will help identify the most effective and least invasive surgical approach for treating this condition.
- Long-term outcomes: There is a need for studies that examine the long-term outcomes of surgical intervention for gynecomastia. This will help determine the effectiveness of different surgical techniques in preventing recurrence and achieving satisfactory cosmetic results.
- Patient selection criteria: Studies are needed to identify the appropriate patient selection criteria for different surgical techniques. This will help surgeons determine which patients are most likely to benefit from a particular surgical approach and avoid unnecessary complications.
- Psychological impact: Further research is needed to examine the psychological impact of gynecomastia and the effectiveness of different surgical interventions in improving patients’ quality of life and psychological well-being.
- Hormonal management: Studies are needed to examine the effectiveness of hormonal management in treating gynecomastia and reducing the need for surgical intervention. This will help identify non-surgical treatment options for patients who may not be suitable candidates for surgical intervention.
- These were the starting group of patients while developing the new technique. We believe with more experience we are achieving better outcomes so we plan to compare another group of patients with this study group to confirm our thoughts regarding better outcomes.

### Electronic supplementary material

Below is the link to the electronic supplementary material.

Additional File 1: Satisfaction Questionnaire: This supplementary file contains a satisfaction questionnaire that was administered to the participants in our study. The questionnaire aimed to assess various aspects related to patient satisfaction following the surgical intervention for gynecomastia.

## Data Availability

All data generated or analyzed during this study are included in this published article and its supplementary information file.

## Abbreviations

NAC: nipple areolar complex
VASER: vibration amplification of sound energy at resonance
