# Supplementary material for: Liposuction and port site nipple sparing mastectomy: an alternative method for the operative treatment of gynecomastia at Alexandria main university hospital
Source: BMC Surg. 2023 Aug 21;23:244. doi: 10.1186/s12893-023-02146-9 (PMC10441726; doi:10.1186/s12893-023-02146-9)
Supplement: Supplementary file 1 — Additional File 1: Satisfaction Questionnaire: This supplementary file contains a satisfaction questionnaire that was administered to the participants in our study. The questionnaire aimed to assess various aspects related to patient satisfaction following the surgical intervention for gynecomastia. [file 12893_2023_2146_MOESM1_ESM.docx]

Results from the questionnaire:

1- scar : only one patient complained if drain site scar shape

2- hypothesia: 72 patients reported hypothesia

3- asymmetry: none

4- satisfaction rate out of 10:

6 patients reported 4 or below ( not satisfied )

97 patients 8 or more ( satisfied )

5- Do you think of revision of surgery ?

6 out of 97

The questionare was

- Do yoy have any problems with the lateral scar ? (Yes/no)

- Do you feel any nipple hypothesia ? (Yes/no)

- Do you feel any asymmetry ? (Yes/no)

- How are you satisfied out of 10 ? ( 4 or less not satisfied , 8 or more satisfied )

- Do you think you need a revision surgery now or later ?

(Yes/no )
